# Impact of pain on mental effort assessed as cardiovascular reactivity

**DOI:** 10.1097/PR9.0000000000000917

**Published:** 2021-04-01

**Authors:** Tamara Cancela, Nicolas Silvestrini

**Affiliations:** Department of Psychology, University of Geneva, Geneva, Switzerland

**Keywords:** Effort, Pain, Motivation, Cardiovascular reactivity

## Abstract

**Introduction:**

Pain typically impairs task performance, increases fatigue, and is associated with behavioral disengagement. Together, this suggests that pain impacts effort, defined as the mobilization of resources to carry out behavior. However, empirical evidence on this issue is lacking.

**Objectives:**

Drawing on motivational intensity theory, this study investigated the impact of pain on effort mobilization during cognitive task performance.

**Methods:**

We administered individually adjusted painful thermal stimulations during an easy memory task during which we measured effort as cardiovascular reactivity—a valid and objective measure. Control conditions included painful stimulations alone, the memory task alone, and the memory task with nonpainful heat stimulations. We expected pain to increase subjective difficulty due to additional demand on cognitive functioning, and in turn effort according to motivational intensity theory. Effort-related cardiovascular reactivity was predicted to increase along these conditions: pain-alone, task-alone, task with nonpainful stimulations, and the strongest effort when painful stimulations were administered during the task.

**Results:**

Overall, the findings offered support to our hypotheses. As predicted, results showed that pain increased subjective task difficulty (*P* = 0.020). Moreover, most of the effort-related cardiovascular measures showed the expected pattern with the strongest reactivity when painful stimulations were administered during the task compared with the other conditions (*p* < 0.02).

**Conclusion:**

These results are first evidence that pain impacts mental effort assessed as cardiovascular reactivity. We discuss how such extra effort might be related to the persistent feeling of fatigue and behavioral disengagement frequently observed in patients with chronic pain.

## Abstract 2

Supplemental Digital Content is Available in the Text.

Pain induced by noxious thermal stimulations increases subjective task difficulty and effort-related cardiovascular response during the performance of a memory task.

## 1. Introduction

Patients with chronic pain often report a persistent feeling of fatigue.^6,7,10,42,44^ Moreover, it is common for these patients to disengage from daily activities,^44,46^ which potentially contributes to the maintenance of pain.^46^ A recent theoretical framework suggests that fatigue and behavioral disengagement in the context of pain are related to the construct of effort,^44^ which is here defined as the amount of resources mobilized for goal achievement.^16^ However, this issue has not been experimentally tested so far. This study aimed to test the impact of pain on effort-related cardiovascular reactivity in the context of cognitive task.

To draw our predictions, we build on motivational intensity theory,^3^ which predicts that effort is determined by subjective task difficulty as long as success is possible and effort justified. Accordingly, following a resource conservation principle, people mobilize low effort if they perceive a task as easy and stronger effort if they perceive a task as difficult. However, if a task is perceived as too difficult or if the required effort is not justified by success importance, effort mobilization should be low—ie, disengagement.

Wright^49^ integrated these predictions with Obrist's active coping approach^30^ assuming that effort is mediated by sympathetic beta-adrenergic discharge to the myocardium.^30,31^ Cardiac pre-ejection period (PEP) is the most sensitive cardiovascular parameter to beta-adrenergic activity because it reflects cardiac contractility.^1,38^ Moreover, systolic blood pressure (SBP) is also influenced by cardiac contractility through its impact on cardiac output^15,51^ and is commonly used as an indicator of effort.^17,18,34,49,51^ By contrast, Obrist found low sympathetic activity when individuals have no control over the situation such as during inescapable painful stimulations suggesting low-effort mobilization during passive coping.^31,32^ Wright's paradigm^49^ was supported in numerous studies testing the influence of distinct variables on mental effort^35^ (see Effort-related Cardiovascular Measures, supplemental digital content 1, for an example of a study, available at http://links.lww.com/PR9/A98), including the influence of priming words related to the concept of pain.^39,40^ However, no study tested this effect with physical pain to this day.

Previous studies indicated that pain can impair concurrent task performance,^5,21^ although such effects seem to occur only during demanding executive tasks and not simple tasks.^20,28,29^ Overall, this suggests that pain and cognitive functioning share common and limited processes.^5,22^ Accordingly, pain leads to additional demand on cognitive functioning presumably due to its negative affective component^27^ and its propensity to capture attention.^8,9,22,45^ Together, these findings suggest that pain increases subjective task difficulty, which should in turn influence effort mobilization according to motivational intensity theory.^3^

We tested these predictions in the present experiment by administering painful thermal stimulations during an easy memory span task while we assessed effort-related cardiovascular reactivity. Control conditions included painful stimulations alone, task alone, and task with nonpainful heat stimulations. We predicted that subjective difficulty and effort-related cardiovascular reactivity should increase linearly along these conditions: pain-alone, task-alone, task with nonpainful stimulations, and task with painful stimulations.

## 2. Method

### 2.1. Participants and design

Thirty volunteers (15 women and 15 men; mean age 21.5 years) were recruited by announcement at the University of Geneva and received 40 Swiss Francs (about 40 USD) for their participation. Participants were free of self-reported acute or chronic pain, and cardiovascular, neurological, or psychological disease. The study followed a within-subject design with 4 conditions: *pain-alone*, *task-alone*, task with nonpainful heat stimulations (*task-warmth*), and task with painful stimulations (*task-pain*). Moreover, participants were assigned to 1 of 4 condition orders (see below). We estimated the sample size based on a power analysis assuming a medium effect size (Cohen *d* = 0.5, *f* = 0.25) with a power of 0.80 and an alpha of 0.05, which led to an estimated sample of about 20 participants. We complied with relevant ethical regulation, and the regional ethical committee in Geneva approved the study protocol (see Ethics Statement, supplemental digital content 1, available at http://links.lww.com/PR9/A98, for more details).

### 2.2. Material

The procedure was computerized with a script running in MATLAB using the Psychtoolbox.^2^

#### 2.2.1. Thermal stimulations

We administered painful and nonpainful heat stimulations with a computer-controlled thermal stimulator and a 25 × 50-mm fluid-cooled Peltier thermode (MSA Thermotest, Somedic SenseLab, Sösdala, Sweden). The thermode was placed on the left side of the participant's left leg below the middle of the tibia. The baseline temperature was set to 36°C and each stimulation lasted 16 seconds (3 seconds of temperature increase, 10 seconds of plateau, and 3 seconds of temperature decrease). The thermode delivered heat stimulations ranging from 39 to 48.5°C.

#### 2.2.2. Cognitive task

**Figure 1.**
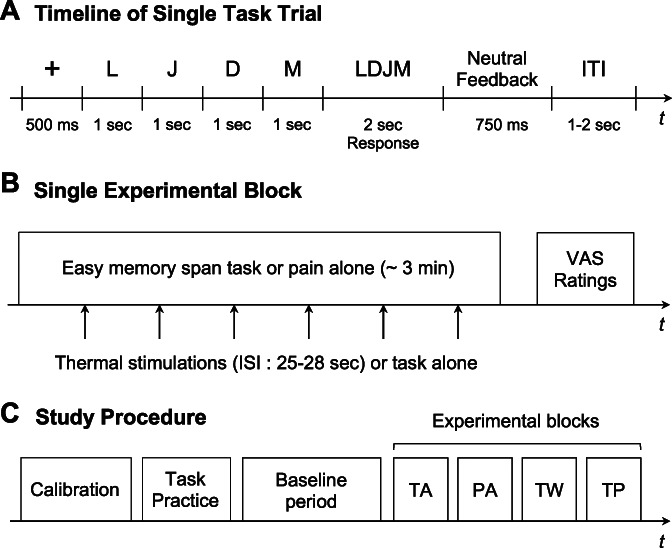
Timeline of a single task trial (A), overview of a single experimental block (B), and overview of the study protocol (C). The order of the experimental conditions was counterbalanced across subjects. Neutral feedback: “Response recorded” or “Please answer more quickly” in case of no response. ISI, interstimulus interval; ITI, intertrial interval; PA, pain-alone; TA, task-alone; TW, task-warmth; TP, task-pain; VAS, visual analogue scale.

We used an easy memory span task.^47^ Four letters were presented individually on the screen (eg, A-B-C-D) and were followed by 4 letters presented simultaneously (the target; eg, ACDB). Participants had to determine if the target was in the same order as the 4 individually presented letters to remember. Participants had to press a green or red key on the keyboard for “yes” (eg, ABCD) and “no” responses (eg, ACDB), respectively. The timing and the details of a single task trial are presented in Figure 1A. Sixty different items were randomly divided into the 3 task blocks for each participant (20 trials per block).

#### 2.2.3. Physiological measures

We assessed PEP (in milliseconds [ms]) by measuring electrocardiogram and impedance cardiogram (ICG) signals using a CardioScreen 2000 system (Medis, Ilmenau, Germany). Two pairs of electrodes were placed on the left side of the base of the participant's neck and on the left middle axillary line at the level of the xiphoid. The resulting signals were sampled at 1000 Hz. Moreover, we assessed SBP (in millimeters of mercury [mm Hg]) using a NIBP SunTech module (Medis, Ilmenau, Germany) integrated within the CardioScreen 2000 and that uses oscillometry. A blood pressure cuff was placed over the brachial artery above the elbow of the participant's left arm and was automatically inflated in 1-min intervals.

In addition, we also measured heart rate (HR; in beats per minute) and diastolic blood pressure (DBP; in millimeters of mercury [mmHg]) to get a fuller picture of cardiovascular reactivity (see Additional Physiological Measures, supplemental digital content 1, for more details about HR and DBP measures, available at http://links.lww.com/PR9/A98).

### 2.3. Procedure

The study was run in individual sessions, which took about one and a half hours. After signing an informed consent form and answering demographic questions (age, sex, and medical history), participants took a seat in a comfortable chair in front of a computer. The experimenter then applied the electrodes, the blood pressure cuff, and the thermode on the participant.

Participants started with a calibration period to individually adjust the temperature of the painful and nonpainful heat stimulations (see Calibration Procedure, supplemental digital content 1, for more details, available at http://links.lww.com/PR9/A98). In the main experiment, we consistently delivered the same temperature for the respective painful and nonpainful conditions.

Then, participants received the instructions for the memory span task and performed 10 practice trials with correctness feedback. After these practice trials, the experimenter left the participant alone and went to an adjacent room.

The experiment started with a cardiovascular baseline assessment period (8 minutes). This consisted of recording cardiovascular activity while participants watched a hedonically neutral documentary film. Cardiac activity was continuously assessed and blood pressure was measured every minute.

Then, participants went through the 4 experimental conditions, which were counterbalanced according to a Latin square design. Each condition lasted 3 minutes during which blood pressure was assessed every minute and cardiac activity was continuously measured. In the *pain-alone* condition, participants received 6 painful heat stimulations occurring approximately at 26, 52 seconds, 1 minute 18 seconds, 1 minute 44 seconds, 2 minutes 10 seconds, and 2 minutes 36 seconds after block onset (range of interstimulus intervals = 24.75–27.75 seconds). At the end of the block, participants rated average pain intensity associated with these painful stimulations using a visual analogue scale. An overview of a single experimental block is presented in Figure 1B. In the *task-alone* condition, participants performed 20 trials of the memory span task of easy difficulty without any thermal stimulation. After the task, we assessed subjective task difficulty (1 = not at all difficult, 7 = extremely difficult) and subjective ability (1 = not able at all, 7 = extremely able) on 7-point scales. The *task-warmth* condition included 6 nonpainful heat stimulations occurring every 3 trials of the memory span task (a similar timing than the pain alone condition), which was followed by an assessment of pain, subjective task difficulty, and subjective ability as described above. In the *task-pain* condition, participants performed the task while receiving 6 painful stimulations. Between each condition, participants had a pause of about 30 seconds. An overview of the study procedure is presented in Figure 1C.

At the end of the last experimental block, the apparatus was removed and the participants were thanked and received their remuneration.

### 2.4. Data analysis

Electrocardiogram and ICG signals were analysed with Bluebox 2 (version 1.22), an in-house program developed by our laboratory.^33^ R-peaks were identified using a threshold peak-detection algorithm and visually confirmed.^24^ The first derivative of the change in thoracic impedance was computed, and the resulting dZ/dt signal was ensemble averaged over periods of 1 minute using the detected R-peaks.^19^ R-onset and B-point were automatically scored for each artefact-free ensemble average. B-point location was estimated based on the RZ interval as proposed by Lozano et al.^26^ In a second step, B-points were visually inspected and corrected if necessary as recommended.^38^ Pre-ejection period was determined as the time interval (in ms) between the electrocardiogram R-peak onset and the ICG B-point.^1^ We tested our hypotheses on PEP determined by the visually inspected and corrected B-points, and on PEP determined by the automatically detected B-point without correction, which is labelled later on *PEP Lozano*. It is of note that shorter PEP indicates greater beta-adrenergic impact on the heart and therefore stronger reactivity in terms of effort intensity. HR (in beats per minute) was determined by means of the Bluebox 2 as well.^33^

Statistical analysis of the data was performed using STATISTICA software package (version 13.1, StatSoft Inc, Tulsa, OK). We ran preliminary repeated-measures analyses of variance on the baseline PEP and SBP cardiovascular measurements to determine how to compute baseline scores for PEP, SBP, DBP, and HR (see Cardiovascular Baselines, supplemental digital content 1, for more details, available at http://links.lww.com/PR9/A98). Then, cardiovascular change (delta-) scores were calculated for each participant and for each cardiovascular measure by subtracting the baseline scores from the 1-min scores obtained during the experimental conditions.^25^

Extreme values and outliers were recoded using winsorized means (*k* = 5).^23,43^ We replaced cardiovascular reactivity scores above the 95th and below the fifth percentile by the value of the observations at the 95th and the fifth percentile, respectively. We tested for baseline, sex, and order effects (see Baseline, Sex, and Order Effects, supplemental digital content 1, for more details, available at http://links.lww.com/PR9/A98). However, including these variables in the main analyses did not change the results. Therefore, these variables were not further considered.

We applied contrast analyses to test our theory-based predictions (see Statistical Procedure, supplemental digital content 1, for more details, available at http://links.lww.com/PR9/A98). These analyses are the most powerful and most appropriate statistical tool to test predicted patterns of means.^12,37,48^ We predicted a linear increase of effort-related cardiovascular response through the following conditions: low effort in the *pain-alone* condition (contrast weight −3), slight effort in the *task-alone* condition (contrast weight −1), more effort in the *task-warmth* condition (contrast weight +1), and the highest effort in *the task-pain* condition (contrast weight +3; see Justification of Contrast Weights, supplemental digital content 1, for a detailed theoretical justification of these contrast weights, available at http://links.lww.com/PR9/A98).

To control whether the expected pattern was stable or varied along the task, we investigated potential interaction with time (3 minutes per block) by testing Contrast × Time interactions for each cardiovascular measure. We analyzed time effects using a linear contrast depicting a decrease in cardiovascular reactivity from the first to the last minute of the task—a typical finding with cardiovascular measures due to habituation or learning.^11,41^ Given the directed predictions, these analyses were one-tailed. In case of significant interaction, we tested our contrast on cardiovascular reactivity during each minute constituting the experimental blocks to determine whether the expected pattern was supported on each minute separately.

Based on our hypotheses, we also used linear contrasts to analyse subjective difficulty and ability ratings. We expected an increase in subjective difficulty and a decrease in ability ratings through the following conditions: *task-alone*, *task-warmth*, and *task-pain*. Moreover, regarding task performance and based on previous findings,^5^ we also expected more errors and slower responses through the following conditions: *task-alone*, *task-warmth*, and *task-pain*, due to additional cognitive demand provided by the nonpainful distractors and pain, respectively. Finally, regarding pain ratings and based on previous findings,^5^ we expected lower pain in the *task-pain* condition compared with the *pain-alone* condition due to the potential distraction provided by the task, and the weakest pain in the *task-warmth* condition. Therefore, task performance and pain ratings were analysed with linear contrasts as well. In case of significant linear contrasts, we applied one-tailed tests for follow-up cell comparisons. The alpha-error level for all tests was fixed at 0.05.

## 3. Results

### 3.1. Thermal stimulation

The calibration procedure determined a mean temperature of 46.1°C for the painful stimulation (SD = 0.90, range = 44.5–48.5) and 41.9°C for the nonpainful stimulation (SD = 1.11, range = 39–44.5).

### 3.2. Pre-ejection period reactivity

The a priori linear contrast was not significant for PEP reactivity, *t*(29) = 1.13, *P* = 0.134, η^2^ = 0.04, whereas the Contrast × Time interaction was significant, *t*(29) = 2.53, *P* = 0.009, η^2^ = 0.18. This interaction emerged because the contrast was not significant during the first and the second minutes (*p* > 0.31), but it was during the third minute, *t*(29) = 2.58, *P* = 0.008, η^2^ = 0.19. However, the pattern of cell means during the third minute did not correspond to our predictions. Cell means and standard errors were as follows: *pain-alone* (M = −0.99, SE = 0.49); *task-alone* (M = −0.51, SE = 0.49); *task-warmth* (M = 0.50, SE = 0.49); and *task-pain* (M = 0.10, SE = 0.66).

### 3.3. Pre-ejection period Lozano reactivity

**Figure 2.**
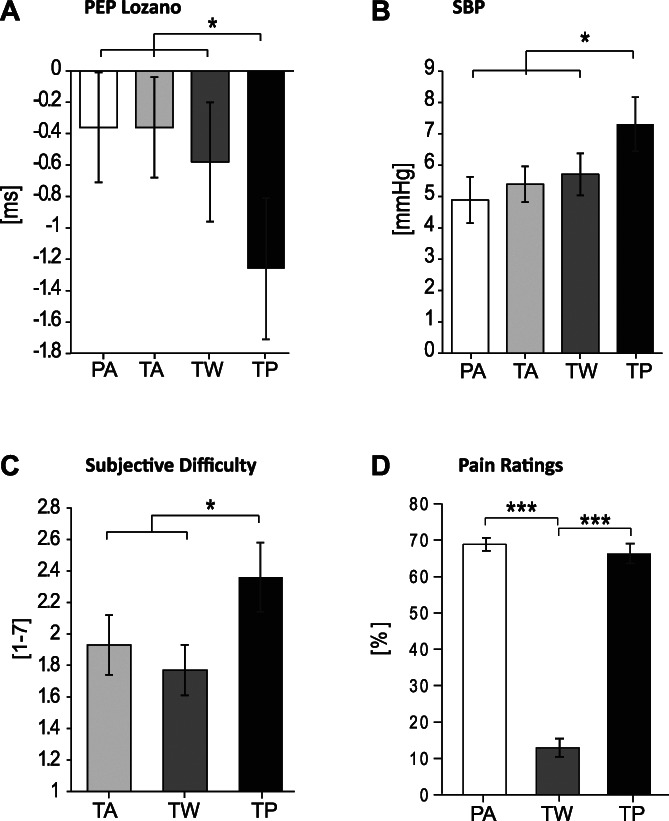
Means and SEs of automatically detected pre-ejection period reactivity (PEP Lozano, A) during the first minute of the task, systolic blood pressure reactivity (SBP, B) during the whole task, subjective task difficulty (C), and pain ratings (D). *p < 0.05; ***p < 0.001. PA, pain-alone; TA, task-alone; TW, task-warmth; TP, task-pain.

The a priori linear contrast was also not significant for PEP Lozano reactivity, *t*(29) = 1.64, *P* = 0.056, η^2^ = 0.09, whereas the Contrast × Time interaction was significant, *t*(29) = 2.93, *P* = 0.004, η^2^ = 0.23. Here, this interaction emerged because the contrast was significant during the first minute of the task, *t*(29) = 2.69, *P* = 0.006, η^2^ = 0.20, and not during the second and third minutes (*p* > 0.10). The pattern of cell means largely supported our predictions (Fig. 2A). Hypothesized contrasts revealed stronger reactivity in the task-pain condition as compared to the task-warmth, *t*(29) = 2.10, *P* = 0.023, η^2^ = 0.13, the task-alone, *t*(29) = 2.25, *P* = 0.016, η^2^ = 0.15, and the pain-alone, *t*(29) = 2.81, *P* = 0.005, η^2^ = 0.21, conditions. Other comparisons were not significant (*p* > 0.09).

### 3.4. Systolic blood pressure reactivity

The a priori linear contrast was significant for SBP reactivity, *t*(29) = 3.17, *P* = 0.002, η^2^ = 0.26, whereas the Contrast × Time interaction was not (*P* = 0.463). The pattern of SBP reactivity also supported our predictions (Fig. 2B). Hypothesized contrasts revealed that SBP reactivity was stronger in the *task-pain* condition than in the *task-warmth, t*(29) = 2.05, *P* = 0.025, η^2^ = 0.13, the *task-alone*, *t*(29) = 2.22, *P* = 0.017, η^2^ = 0.15, and the *pain-alone, t*(29) = 3.25, *P* = 0.003, η^2^ = 0.27, conditions. No significant difference emerged among these 3 latter conditions (*p* > 0.12).

### 3.5. Diastolic blood pressure and heart rate reactivity

DBP and HR reactivity showed the expected pattern as well (see DBP and HR Results, supplemental digital content 1, for the details, available at http://links.lww.com/PR9/A98).

### 3.6. Subjective task difficulty and ability ratings

The linear contrast was significant for subjective task difficulty, *t*(29) = 2.16, *P* = 0.020, η^2^ = 0.14. As predicted, hypothesized contrasts indicated stronger difficulty ratings in the *task-pain* condition compared with the *task-warmth, t*(29) = 3.38, *P* < 0.001, η^2^ = 0.28, and the *task-alone*, *t*(29) = 2.16, *P* = 0.020, η^2^ = 0.14, conditions (Fig. 2C). No significant difference emerged between these 2 latter conditions (*P* = 0.086). Subjective ability ratings supported our predictions as well (see Subjective Task Ability Results, supplemental digital content 1, for the details, available at http://links.lww.com/PR9/A98).

### 3.7. Task performance

The linear contrast was not significant for the percentage of correct responses, *t*(29) = 1.41, *P* = 0.085, η^2^ = 0.06. Cell means and standard errors were as follows: *task-alone* (M = 94.17, *SE* = 1.02), *task-warmth* (*M* = 94.17, *SE* = 1.05), and *task-pain* (*M* = 95.83, *SE* = 0.72).

Moreover, the linear contrast was not significant for reaction times, *t*(29) = 0.78, *P* = 0.221, η^2^ = 0.06. Cell means and standard errors of mean for reaction time were as follows: *task-alone* (*M* = 921.68, *SE* = 32.59); *task-warmth* (*M* = 927.66, *SE* = 31.47); and *task-pain* (*M* = 908.99, *SE* = 31.95).

### 3.8. Pain ratings

The linear contrast was highly significant for pain ratings, *t*(29) = 19.06, *P* < 0.001, η^2^ = 0.92*.* Hypothesized contrasts revealed lower pain in the *task-warmth* condition (*M* = 12.87, *SE* = 2.48) compared with the *task-pain*, *t*(29) = 15.30, *P* < 0.001, η^2^ = 0.89 and the *pain-alone, t*(29) = 19.06, *P* < 0.001, η^2^ = 0.92, conditions (Fig. 2D). No significant difference emerged between these 2 latter conditions (*P* = 0.085).

## 4. General discussion

Overall, these findings indicate the first evidence for our hypotheses that pain increases subjective task difficulty and impacts effort during cognitive performance. We obtained significant effects on subjective task difficulty and some of our effort-related cardiovascular measures (SBP, DBP, and HR) in line with our predictions. By contrast, PEP Lozano only supported our predictions during the first minute of the task, whereas corrected PEP did not show the expected pattern.

As predicted, the results showed an impact of pain on subjective task difficulty. Participants perceived the task as more difficult and felt less able to succeed in the task when they received painful stimulation than when they performed the task without stimulation. These findings are consistent with previous studies suggesting that pain requires the allocation of additional cognitive resources during task performance.^5,22^ The primary function of pain is warning about potential threats to the body.^9^ To achieve this function, pain has an inherent negative affective component^27^ and the ability to capture attention,^9,22,45^ which leads pain to act as a potent distractor. This is expected to consequently increase subjective difficulty and effort, which may compensate for potential task performance decrements.^36^

Moreover, cardiovascular reactivity offered some support to our predictions on effort. Drawing on motivational intensity theory, we predicted that subjective task difficulty should determine effort and we expected an increase in effort along the following conditions: *pain-alone*, *task-alone*, *task-warmth*, and *task-pain*. This study showed the anticipated pattern of cardiovascular reactivity for most of the cardiovascular measures. The expected linear trend emerged for PEP Lozano reactivity during the first minute of the task, and for SBP, DBP, and HR reactivity consistently for the whole task. For these measures, we found stronger reactivity during task performance when participants received painful stimuli compared with the 3 other conditions. Therefore, these findings are the first direct evidence that pain increases effort during cognitive performance. As suggested by a recent theoretical account on the influence of pain on fatigue,^44^ pain-related task interference leads to increased effort and gradually induces fatigue. In the present experiment, we did not assess fatigue but our findings support the hypothesis of an effect of pain on effort.

Findings related to task performance did not reveal any effect of our manipulations on task performance. Moreover, our prediction about the analgesic effect of cognitive performance on pain was not found. It is plausible that the task was not difficult enough to show these effects.^20,28,29^ However, it is also possible that undiscovered confounding factors affect these issues, and effort might actually be one of them. In line with this idea, extremely difficult tasks should lead to disengagement,^3^ ie, no effort, which might reduce the reciprocal influence of task and pain. Moreover, increased effort during feasible tasks may compensate for performance detriments as suggested previously.^36^ Therefore, future studies might further investigate the role of effort in the relationship between pain and task performance.

As discussed above, these findings shed light on the impact of pain on fatigue observed in the context of chronic pain.^44^ Coping with pain during daily activities calls for extra effort due to increased difficulty, which should lead to a persistent feeling of fatigue. However, as mentioned in the introduction, it is also often reported that patients with chronic pain disengage from daily activities.^44,46^ Here, motivational intensity theory also offers clear predictions about this disengagement. If the required effort exceeds the effort that is justified by success importance, people should disengage. Accordingly, it is plausible that patients with chronic pain disengage from some activities because they perceive them as too difficult and requiring too much effort. Therefore, as mentioned in the theoretical account on fatigue,^44^ pain treatment protocol might consider helping patients selecting valued but feasible goals. Furthermore, patients may benefit from self-regulatory strategies such as the use of action plans or implementation intentions to reduce effort in goal pursuit. To follow-up on these ideas, our research program plans future studies to experimentally test these predictions about disengagement drawing on motivational intensity theory.

As limitations, it is worth noting that the expected pattern for cardiovascular reactivity was only visible for the automatically detected PEP (PEP Lozano) and not for the visually corrected PEP. We do not have a clear explanation for this discrepancy. It seems that the PEP Lozano pattern more closely matched the pattern of the other cardiovascular parameters (SBP, DBP, and HR). Moreover, given that HR and DBP reactivity did not decrease together with PEP Lozano, the effects on PEP Lozano are hardly explainable in terms of preload or afterload effects and rather reflect sympathetic activity.^38^ As a second limitation, the pattern only emerged during the first minute of the task for PEP Lozano reactivity. This finding suggests that sympathetic activity and effort were stronger at the beginning of the task and then reduced due to habituation or learning. Given that PEP Lozano is a very sensitive measure of sympathetic activity, it seems that the anticipated effect was only visible during the first minute. By contrast, we found the predicted effect during the whole task for SBP, a reliable indicator of effort as well, and for DBP and HR. Therefore, despite these limitations, there is evidence for an impact of pain on effort-related cardiovascular reactivity in accordance with our predictions.

To conclude, this study showed that pain has an impact on subjective difficulty and effort assessed as cardiovascular reactivity. These findings extend the large body of research—more than 100 studies—drawing on motivational intensity theory and the integrative approach of Wright.^3,49^ In this context, it is of note that previous work revealed similar effects on effort with mood, depression, or fatigue.^4,13,14,50^ This suggests that effort and self-regulation might be common between several domains such as depression and chronic pain. Actually, one may consider the ability to exert effort to be somewhat limited. Failure to inhibit self-regulatory fatigue may represent a risk factor for various pathological conditions including chronic pain, which would be consistent with effects of pain on executive tasks.^42^ Altogether, this highlights the central role of effort and self-regulation in the context of pain and calls for further research on this topic.

## Disclosures

The authors have no conflicts of interest to declare.

## Supplementary Material

SUPPLEMENTARY MATERIAL

## Appendix A. Supplemental digital content

Supplemental digital content associated with this article can be found online at http://links.lww.com/PR9/A98.
