# Supplementary material for: Impact of pain on mental effort assessed as cardiovascular reactivity
Source: Pain Rep. 2021 Apr 1;6(1):e917. doi: 10.1097/PR9.0000000000000917 (PMC8104428; doi:10.1097/PR9.0000000000000917)
Supplement: SUPPLEMENTARY MATERIAL [file painreports-6-e917-s001.pdf]

The impact of pain on mental effort assessed as cardiovascular reactivity

Tamara Cancela & Nicolas Silvestrini

Supplemental digital content 1

Effort-related cardiovascular reactivity. Wright [22] integrated the predictions of motivational intensity theory [1] with Obrist's active coping approach [13] and posited that beta-adrenergic sympathetic influence on the heart is proportional to subjective task demand as long as success is possible and the required effort is justified. Numerous studies supported these predictions, as reviewed elsewhere in detail [9,10,17,24]. For instance, Richter and colleagues [15] assigned participants to one of four task difficulty conditions (easy, moderate, difficult and impossible) following a between-subject design. They assessed pre-ejection period (PEP), systolic blood pressure (SBP), diastolic blood pressure (DBP), and heart rate (HR) during a baseline period (8 min) and task performance (5 min). Cardiovascular reactivity was computed as change scores by subtracting the baseline scores from the respective task scores. Results showed that PEP and SBP reactivity was lower in the easy, stronger in the moderate and the strongest in the difficult task. In the impossible condition, PEP and SBP reactivity was low, which reflected disengagement as predicted by motivational intensity theory. Therefore, these and other findings supported the hypothesis that beta-adrenergic sympathetic impact on the heart reliably reflects effort mobilization and that cardiovascular reactivity is a valid and objective measure of effort.

According to motivational intensity theory, success importance directly determines effort only when task difficulty is unfixed, unknown or unspecified. When task difficulty is fixed and known, the theory predicts that success importance determines the upper limit of the relationship between subjective task demand and effort mobilization, i.e. the maximally justified effort (see [1,17,20,22], for a detailed presentation of the theory).

Ethics statement. The procedure was conducted in accordance with the Declaration of Helsinki, the principles of Good Clinical Practice, the Human Research Act and the Human Research Ordinance.

*Additional physiological measures***.** It is of note that DBP and HR parameters are less sensitive to beta-adrenergic impact on the heart than PEP or SBP and therefore to effort according to Wright's integration [8]. HR is influenced by both sympathetic and parasympathetic activity and DBP is more strongly influenced by peripheral resistance than SBP [12]. However, HR and DBP may also respond together with PEP and SBP [2,6,7,16,23,25,26]. This may occur for HR when the sympathetic impact is stronger than the parasympathetic impact and for DBP when the sympathetic impact on cardiac contractility is strong enough to override the impact of peripheral resistance. Moreover, HR and DBP should always be assessed together with PEP to control for pre-load and after-load effects on PEP reactivity [19].

Calibration procedure. The calibration consisted of gradually increasing and decreasing the temperature of the stimulations three times. The first stimulation started at a temperature of 39°C and the following stimulations' temperatures increased by steps of 2° until the participant rated pain as more than 6 out of 10 on a visual analogue scale (VAS; (0) *no sensation*, (1) *non-painful warmth*, (2) *just painful*, (5) *moderate pain*, (8) *strong pain* and (10) *the strongest pain*). At this point, temperature decreased by steps of 2° until the participant rated pain as less than 1 out of 10. In the second back and forth, temperature increased by steps of 1° until pain ratings reached 6 out of 10 and decreased until pain ratings were at 4 out of 10. In the last one, temperature increased and decreased by steps of 0.5°C following the same turning point rules as the second back and forth. For each participant, we created a scatterplot displaying the administered temperature on the x-axis and pain responses on the y-axis. By visual inspection, we selected the painful temperature as the temperature eliciting a pain response of about 6 out of 10— i.e. a moderately strong pain. We selected the non-painful temperature as eliciting a pain

response of about 1 out of 10, which suggests a non-painful heat but still perceptible. We confirmed this selection with a single trial for both kinds of stimulations, which was followed by a pain rating.

Cardiovascular baselines. Preliminary repeated measures ANOVAs on the baseline PEP and SBP cardiovascular measurements indicated a main effect of time for both measures (ps < .001). These effects emerged because baseline scores decreased from the first to the fifth minute and then remained stable. Therefore, PEP, SBP, DBP and HR baseline scores were calculated as the arithmetic mean of the last three minutes of the baseline period. Means of baseline scores were as follows: PEP (M = 104.92, SE = 2.32), PEP Lozano (M = 105.66, SE = 1.29), SBP (M = 113.88, SE = 2.26), DBP (M = 69.04, SE = 1.43) and HR (M = 75.16, SE = 2.01).

Baseline, gender, and order effects. To control whether baseline assessment had an impact on reactivity measures, repeated measures ANCOVAs were applied with the baseline measures as covariates. There were neither covariate main effects on reactivity measures (ps > .54) nor interactions (ps > .26) between the covariates and the experimental conditions, except for a trending interaction for SBP (p = .085). However, including SBP baseline scores as a covariate did not change the main contrast findings on SBP reactivity (p = .001 with covariate, p = .002 without covariate). Therefore, baseline scores were not further considered in the subsequent analysis for the sake of parsimony.

Moreover, additional preliminary 2 (gender) x 4 (condition) repeated measures ANOVA did not show any main effect (ps > .15) or interaction (ps > .27) with gender, which was therefore not further considered. We also tested the impact of order as a between-subject variable and results revealed neither main effects of order (ps > .38) nor interactions with reactivity scores (ps > .34), with the exception of two trending interaction effects for SBP (p = .053) and HR (p = .170). However, including order in the analyses did not change the main findings for both SBP (a

priori linear contrast: $p < .001$ when including order as a between-subject variable, $p = .002$ without including order as a between-subject variable) and HR (a priori linear contrast: $p = .001$ when including order as a between-subject variable, $p = .002$ without including order as a between-subject variable). Therefore, order was not further considered in the subsequent analysis for the sake of parsimony.

Statistical procedure. To run contrast analyses on STATISTICA, we selected RM-ANOVA and included the dependent variables to analyze. To test our main contrast on cardiovascular reactivity, we included the four variables associated with the four experimental conditions for a given cardiovascular parameter. Then, we indicated the within-factor (experimental conditions) and the number of levels (4). Finally, we specified the a priori defined contrast weights for each condition, which overall corresponded to a linear contrast along the four conditions (-3, -1, +1, +3). To test Contrast x Time interactions, we included the twelve variables (4 experimental conditions x 3 minutes of the task) for a given cardiovascular parameter. Then, we indicated two within-factors (experimental conditions and time) and their numbers of levels (4 and 3, respectively). Finally, we specified the a priori defined contrast weights for the experimental conditions (-3, -1, +1, +3) and for time (+1, 0, -1), which both correspond to linear contrasts. It is of note that the output stats of such analyses on STATISTICA are *t*-values. Actually, contrasts always have only one degree of freedom in the numerator, which justify providing *t*-values for these analyses. Moreover, providing *t*-values is in line with the rationale of using one-sided test when the direction of the effect is clearly predicted a priori as in the present study.

Justification of contrast weights. We predicted a linear increase of effort-related cardiovascular response through the following conditions: low effort in the *pain-alone* condition (contrast weight -3), slight effort in the *task-alone* condition (contrast weight -1), more effort in the *task-warmth* condition (contrast weight +1) and the highest effort in *the task-pain* condition

(contrast weight +3). Drawing on the work of Obrist [14] showing minimal sympathetic activity in passive coping situation, i.e. when individuals have no control over the painful stimuli, we expected low cardiovascular reactivity in the pain-alone condition. Here, individuals had no task to perform and were passively exposed to painful stimulations. We included this condition to control whether pain had a direct impact on cardiovascular activity. More reactivity was predicted during the *task-alone* condition because participants worked on the easy cognitive task, which should result in a slight increase in effort. We predicted still more reactivity in the *task-warmth* condition due to the distraction provided by the stimulations [5], which should increase subjective task difficulty and in turn effort. Finally, we predicted the highest reactivity in the *task-pain* condition. Given that pain is an unpleasant experience, it should attract attention [3,11,18]. Accordingly, pain represents a warning signal for threatening stimuli that is expected to disrupt cognitive processing acting as a potent distractor [4,11,21]. Therefore, pain should require the allocation of additional cognitive resources during task performance and increase subjective task difficulty and in turn effort.

DBP and HR results. The *a priori* linear contrast was significant for DBP reactivity $t(29) = 3.29$, $p = .002$, $\eta^2 = .27$, whereas the Contrast x Time interaction was not significant ($p = .160$). The pattern of DBP reactivity corresponded to the one of PEP Lozano and SBP reactivity. Hypothesized contrasts indicated that DBP reactivity was stronger in the *task-pain* condition ($M = 5.23$, $SE = 0.60$) than in the *task-alone* ($M = 3.73$, $SE = 0.48$), $t(29) = 3.24$, $p = .002$, $\eta^2 = .27$, and the *pain-alone* ($M = 3.77$, $SE = 0.48$), $t(29) = 2.91$, $p = .004$, $\eta^2 = .23$, conditions. Moreover, DBP reactivity in the *task-warmth* condition ($M = 4.63$, $SE = 0.56$) was stronger than in the *task-alone* condition, $t(29) = 2.19$, $p = .019$, $\eta^2 = .14$. Other comparisons were not significant (*p*s > .08).

Regarding HR reactivity, the a priori linear contrast was significant as well, $t(29) = 3.25$, $p = .002$, $\eta^2 = .27$. The Contrast x Time interaction was also significant, $t(29) = 3.06$, $p = .003$, $\eta^2 =$

.24, but additional analyses revealed significant contrast effects for all three minutes (*p*s < .04). Overall, the pattern of HR reactivity corresponded to our predictions. Hypothesized contrasts revealed weaker reactivity in the *pain-alone* (*M* = 0.45, *SE* = 0.55) than in the *task-alone* (*M* = 1.75, *SE* = 0.47), $t(29) = 2.25$, $p = .016$, $\eta^2 = .15$, the *task-warmth* (*M* = 2.27, *SE* = 0.55), $t(29) = 3.68$, $p = .001$, $\eta^2 = .32$ and the *task-pain* (*M* = 2.10, *SE* = 0.61), $t(29) = 2.96$, $p = .003$, $\eta^2 = .23$, conditions. Other comparisons were not significant (*p*s > .07).

Subjective task ability results. The linear contrast was significant as well, $t(29) = 2.84$, $p = .004$, $\eta^2 = .22$. Hypothesized contrasts revealed lower task ability ratings in the *task-pain* condition (*M* = 5.82, *SE* = 0.22) compared to the *task-alone* (*M* = 6.37, *SE* = 0.18), $t(29) = 2.84$, $p = .004$, $\eta^2 = .22$ and to the *task-warmth* (*M* = 6.41, *SE* = 0.17), $t(29) = 2.54$, $p = .009$, $\eta^2 = .18$, conditions. No significant difference emerged between these two latter conditions ($p = .425$).

References

[1] Brehm JW, Self EA. The intensity of motivation. Annu Rev Psychol 1989;40:109–131.

[2] Brinkmann K, Gendolla GHE. Dysphoria and mobilization of mental effort: Effects on cardiovascular reactivity. Motiv Emot 2007;31:71–82.

[3] Cacioppo JT, Gardner WL. Emotion. Annu Rev Psychol 1999;50:191–214.

[4] Eccleston C, Crombez G. Pain demands attention: A cognitive–affective model of the interruptive function of pain. Psychol Bull 1999;125:356–366.

[5] Forster S, Lavie N. Failures to ignore entirely irrelevant distractors: The role of load. J Exp Psychol Appl 2008;14:73–83.

[6] Frazier BD, Barreto P, Wright RA. Gender, perceptions of incentive value, and cardiovascular response to a performance challenge. Sex Roles 2008;59:14–20.

[7] Gendolla GHE. Effort as assessed by motivational arousal in identity-relevant tasks. Basic Appl Soc Psychol 1998;20:111–121.

[8] Gendolla GHE, Richter M. Effort mobilization when the self is involved: Some lessons from the cardiovascular system. Rev Gen Psychol 2010;14:212–226.

[9] Gendolla GHE, Wright RA, Richter M. Effort intensity: Some insights from the cardiovascular system. In: Ryan R, editor. The Oxford handbook of human motivation.

Oxford library of psychology. Oxford: Oxford University Press, 2012. pp. 420–438.

[10] Gendolla GHE, Wright RA, Richter M. Issues in Motivation Intensity Research. In: Ryan R, editor. The Oxford Handbook of Human Motivation. Oxford University Press, 2019. p. 373.

[11] Legrain V, Van Damme S, Eccleston C, Davis KD, Seminowicz DA, Crombez G. A neurocognitive model of attention to pain: Behavioral and neuroimaging evidence. Pain 2009;144:230–232.

[12] Levick JR. An introduction to cardiovascular physiology. London: Hodder education Digital, 2010.

[13] Obrist PA. Cardiovascular Psychophysiology: A perspective. New York: Plenum Press, 1981.

[14] Obrist PA, Gaebelein CJ, Teller ES, Langer AW, Grignolo A, Light KC, McCubbin JA. The Relationship Among Heart Rate, Carotid dP/dt, and Blood Pressure in Humans as a Function of the Type of Stress. Psychophysiology 1978;15:102–115.

[15] Richter M, Friedrich A, Gendolla GHE. Task difficulty effects on cardiac activity. Psychophysiology 2008;45:869–875.

[16] Richter M, Gendolla GHE. Incentive value, unclear task difficulty, and cardiovascular reactivity in active coping. Int J Psychophysiol 2007;63:294–301.

[17] Richter M, Gendolla GHE, Wright RA. Chapter Five - Three Decades of Research on Motivational Intensity Theory: What We Have Learned About Effort and What We Still Don’t Know. In: Elliot AJ, editor. Advances in Motivation Science. Amsterdam: Elsevier, 2016, Vol. 3. pp. 149–186.

[18] Rozin P, Royzman EB. Negativity bias, negativity dominance, and contagion. Personal Soc Psychol Rev 2001;5:296–320.

[19] Sherwood A, Allen MT, Fahrenberg J, Kelsey RM, Lovallo WR, Van Doornen LJP. Methodological guidelines for impedance cardiography. Psychophysiology 1990;27:1–23.

[20] Silvestrini N, Gendolla GHE. Affect and cognitive control: Insights from research on effort mobilization. Int J Psychophysiol 2019;143:116–125.

[21] Van Damme S, Crombez G, Eccleston C. Coping with pain: A motivational perspective. Pain 2008;139:1–4.

[22] Wright RA. Brehm’s theory of motivation as a model of effort and cardiovascular response. In: Gollwitzer P, Bargh J, editors. The psychology of action: Linking cognition and motivation to behavior. New York, NY, US: Guilford Press, 1996. pp. 424–453.

[23] Wright RA, Junious TR, Neal C, Avello A, Graham C, Herrmann L, Junious S, Walton N. Mental fatigue influence on effort-related cardiovascular response: difficulty effects and extension across cognitive performance domains. Motiv Emot 2007;31:219–231.

[24] Wright RA, Kirby LD. Effort determination of cardiovascular response: An integrative analysis with applications in social psychology. Advances in Experimental Social

Psychology. Academic Press, 2001, Vol. 33. pp. 255–307.

[25] Wright RA, Stewart CC, Barnett BR. Mental fatigue influence on effort-related cardiovascular response: Extension across the regulatory (inhibitory)/non-regulatory performance dimension. Int J Psychophysiol 2008;69:127–133.

[26] Zafeiriou A, Gendolla GHE. Implicit aging: Masked age primes influence effort-related cardiovascular response in young adults. Adapt Hum Behav Physiol 2018;4:1–20.
